# Effect of Marine Collagen Peptides on Physiological and Neurobehavioral Development of Male Rats with Perinatal Asphyxia

**DOI:** 10.3390/md13063653

**Published:** 2015-06-05

**Authors:** Linlin Xu, Wenhong Dong, Jie Zhao, Yajun Xu

**Affiliations:** 1 Department of Nutrition and Food Hygiene, School of Public Health, Peking University, No. 38 Xue Yuan Road, Hai Dian District, Beijing 100191, China; E-Mails: bmuliyong58@126.com (L.X.); monydwh@163.com (W.D.); zhaojie0410@sina.com (J.Z.); 2 Beijing Key Laboratory of Toxicological Research and Risk Assessment for Food Safety, Peking University, No. 38 Xue Yuan Road, Hai Dian District, Beijing 100191, China

**Keywords:** marine collagen peptides, perinatal asphyxia, neurobehavioral development

## Abstract

Asphyxia during delivery produces long-term deficits in brain development. We investigated the neuroprotective effects of marine collagen peptides (MCPs), isolated from Chum Salmon skin by enzymatic hydrolysis, on male rats with perinatal asphyxia (PA). PA was performed by immersing rat fetuses with uterine horns removed from ready-to-deliver rats into a water bath for 15 min. Caesarean-delivered pups were used as controls. PA rats were intragastrically administered with 0.33 g/kg, 1.0 g/kg and 3.0 g/kg body weight MCPs from postnatal day 0 (PND 0) till the age of 90-days. Behavioral tests were carried out at PND21, PND 28 and PND 90. The results indicated that MCPs facilitated early body weight gain of the PA pups, however had little effects on early physiological development. Behavioral tests revealed that MCPs facilitated long-term learning and memory of the pups with PA through reducing oxidative damage and acetylcholinesterase (*AChE*) activity in the brain, and increasing hippocampus phosphorylated cAMP-response element binding protein (*p-CREB)* and brain derived neurotrophic factor (*BDNF*) expression.

## 1. Introduction

Perinatal asphyxia (PA) has been associated with a wide spectrum of short-term or long-term neurobehavioral disorders [1,2,3]. Severe PA often results in spasticity, epilepsy and mental retardation, while milder asphyxia is associated with attention deficit hyperactivity syndrome and minimal brain disorder [4]. PA occurs frequently when delivery is prolonged, despite improvements in perinatal care [5,6,7]. The international incidence has been reported as 2–6/1000 term births, reaching higher rates in developing countries [8]. Except for post-asphyctic hypothermia, there is still a lack of effective therapeutic strategies to treat post-asphyctic encephalopathy.

Bioactive peptides that are present in the amino acid sequence of food proteins have become of particular interest in nutrition and food science in recent decades. These peptides, which are inactive within the sequence of the parent protein, can be released by enzymatic proteolysis, for example during gastrointestinal digestion or during food processing [9]. A wide range of activities of bioactive peptides have been described, including antimicrobial, blood pressure-lowering, cholesterol-lowering, antioxidative, and immunomodulatory effects [10,11]. Moreover, some peptides can influence higher brain functions, such as learning and memory, in humans and animals [12]. Dipeptides and tripeptides can cross through the intestinal wall and enter into the bloodstream directly. It is possible that the small peptides can reach the target organs to exert their beneficial biological effects. However, the bigger peptides are broken down into amino acids and/or small peptides in the gut to be absorbed and exert their function.

With marine species comprising approximately half of the total global biodiversity, the sea offers an enormous resource for novel compounds. In fact, the skin, bones, scales, and residual minced meat of marine animals are considered byproducts of the processing industry, which usually cause waste and pollution. However, these “wastes” are high in protein, which offer a resource of functional peptides [13,14]. During the past five decades, fish proteins have been widely studied and their various multifunctional properties have been well described [15,16]. However, it remains unclear whether bioactive peptides from fish proteins have preventive effects against PA induced neurobehavioral disorders. In recent years, we have focused on the bioactive peptides derived from the byproducts of chum salmon (*Oncorhynchus keta*). We previously found antioxidant and promnestic functions of the peptides derived from the skin collagen protein (named marine collagen peptides (MCPs) of this fish in aged animals [17]. Hence, it is reasonable to hypothesize that the MCPs derived from this fish may be a valuable source of neuroprotective peptides. The purpose of the present study was to determine if these peptides were effective in ameliorating long-term behavioral changes caused by PA.

## 2. Results

### 2.1. Effects of MCPs on Body Weight, and Physiological and Neurobehavioral Development Indexes of Male Rats with PA

**Figure 1:**
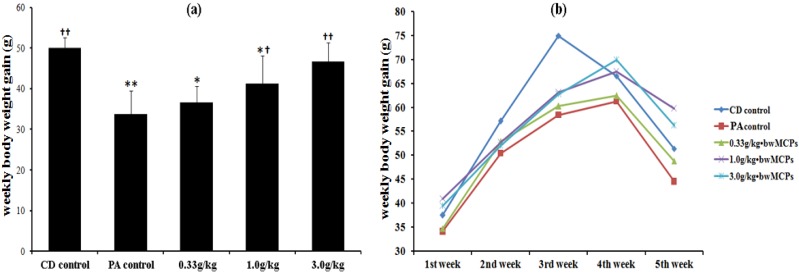
Effect of marine collagen peptides (MCPs) on weekly body weight gain of male rats with PA. (**a**) Weekly body weight gain during the lactation period; (**b**) weekly body weight gain during the first five weeks after weaning. Mean values that were significantly different from those of the CD control group: * *p* < 0.05, ** *p* < 0.01. Mean values that were significantly different from those of the perinatal asphyxia (PA) control group: ^†^ *p* < 0.05, ^††^ *p* < 0.01.

No statistical difference was found in the birth weight of the pups between the groups. However, the mean weekly body weight gain (WBWG, calculated by “the body weight at the end of the week” minus “the body weight at the end of the last week”) during the lactation period was significantly different between the groups (Figure 1a). WBWG of pups in the PA control group was significantly less than that of the non-asphyxia caesarean-delivered (CD) control group (*p* < 0.01). WBWG of pups in 1.0 and 3.0 g/kg body weight MCPs intervention groups were significantly increased compared with the PA control group (*p* < 0.05 and *p* < 0.01, respectively). The after weaning WBWG curve is shown in Figure 1b. Interestingly, the WBWG of the CD control group peaked on the third week after weaning, but peaked on the fourth week in the other PA groups. MCP intervention with 1.0 and 3.0 g/kg showed protective effects, as the WBWG was significantly increased in these two groups compared with the PA control group (*p* < 0.05).

Compared with the CD controls, the early physiological and neurobehavioral developmental indexes were all delayed in the groups with PA. However, MCPs administration did not show significant benefits on these early development parameters (Table 1 and Table 2). 

**Table 1:** Effect of MCPs on early physiological development of male rats with PA.

Groups	*N*	Pinnae Detachment (day)	Incisor Eruption (day)	Eyelid Separation (day)	Testicular Descent (day)
CD control	15	3.500 ± 0.378	11.000 ± 0.267	14.062 ± 0.177	20.125 ± 0.232
PA control	15	4.000 ± 0.267 **	11.938 ± 0.678 **	15.062 ± 0.496 **	20.562 ± 0.417 *
0.33 g/kg	15	3.875 ± 0.232 *	11.750 ± 0.463 **	14.812 ± 0.594 **	20.812 ± 0.458 **
1.0 g/kg	15	3.938 ± 0.177 **	11.625 ± 0.354 *	14.750 ± 0.535 *	20.750 ± 0.463 **
3.0 g/kg	15	3.812 ± 0.259 *	11.562 ± 0.417 *	14.688 ± 0.530 *	20.625 ± 0.354 *

Mean values that were significantly different from those of the CD control group: * *p* < 0.05, ** *p* < 0.01.

**Table 2:** Effect of MCPs on neurobehavioral development of male rats with PA.

Groups	*N*	Surface Righting (day)	Negative Geotaxis (day)	Cliff Avoidance (day)
CD control	15	6.000 ± 0.3120	7.000 ± 0.378	7.062 ± 0.177
PA control	15	6.850 ± 0.5126 **	7.669 ± 0.317 **	7.631 ± 0.344 **
0.33 g/kg	15	6.613 ± 0.3610 *	7.562 ± 0.393 *	7.625 ± 0.354 **
1.0 g/kg	15	6.574 ± 0.3932 *	7.500 ± 0.380 *	7.582 ± 0.397 *
3.0 g/kg	15	6.562 ± 0.4816 *	7.427 ± 0.397 *	7.490 ± 0.373 *

Mean values that were significantly different from those of the CD control group: * *p <* 0.05, ** *p <* 0.01.

### 2.2. Effect of MCPs on the Performance in Behavioral Tests of Male Rats with PA

#### 2.2.1. MCPs Intervention Did Not Significantly Impact the Locomotion of Male Rats with PA in the Open-Field Test

As shown in Table 3, animals with PA stayed much longer in the central cells and crossed fewer grids than animals in the CD control group. Rearing and grooming times were also significantly greater in the PA control group than the CD control group. The parameters in the MCPs administration groups did not show significant differences compared with the PA control. 

**Table 3:** Effect of MCPs on the performance of male rats with perinatal asphyxia in the open field test.

Groups	*N*	Time Spent in Central Grids (s)	Number of Grid Crossing	Frequency of Rearing	Frequency of Grooming
CD control	15	1.50 ± 0.535	57.00 ± 16.449	15.62 ± 6.906	1.88 ± 0.835
PA control	15	3.15 ± 1.309 *	74.25 ± 14.260 *	21.25 ± 5.874	3.12 ± 0.835 **
0.33 g/kg	15	3.12 ± 1.553 *	76.38 ± 12.512 **	21.62 ± 4.406 *	3.00 ± 0.535 **
1.0 g/kg	15	3.12 ± 1.885 *	78.75 ± 11.260 **	22.15 ± 5.365 *	3.00 ± 0.926 **
3.0 g/kg	15	3.14 ± 0.886 *	78.12 ± 13.674 **	21.75 ± 6.112 *	3.00 ± 0.756 **

Mean values that were significantly different from those of the CD control group: * *p* < 0.05; ** *p* < 0.01.

#### 2.2.2. MCPs Improved the Long-Term Spatial Memory of Male Rats with PA in the Morris Water Maze Test

**Figure 2:**
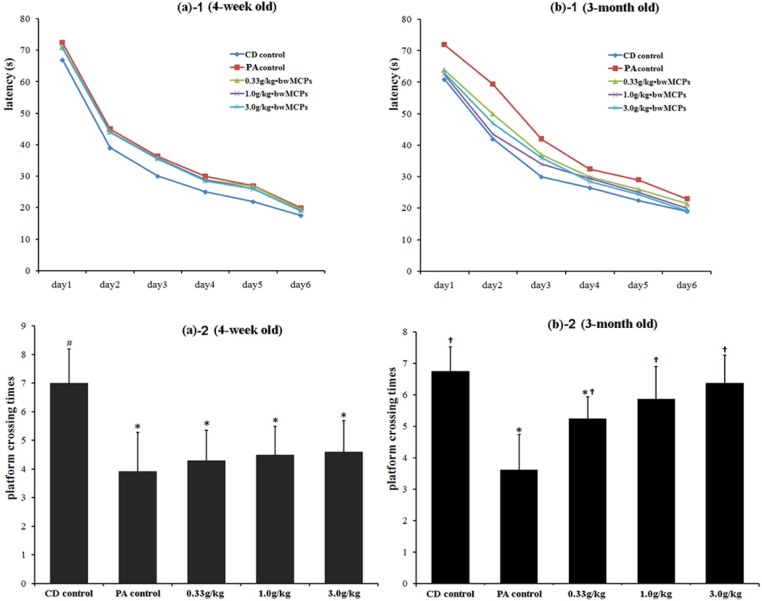
Effect of MCPs on the performance of male rats with PA in the Morris water maze test (*n*=15). (**a**) Results of animals at four weeks of age and (**b**) three months of age. (**1**) The score in the first six days of the training session, and (**2**) the score of platform crossing on the seventh day of the test. Mean values that were significantly different from those of the caesarean-delivered (CD) control group: * *p* <0.05. Mean values that were significantly different from those of the PA control group ^†^ *p* < 0.05.

The Morris water maze test was carried out twice, once at four weeks of age and once at three months of age. During the six-day training sessions, the mean latency to find the submerged platform declined progressively in all the animals. However, it took significantly longer for the animals with PA to locate the platform than those in the CD control group both at four weeks and at three months of age (Figure 2a-1,b-1). In addition, the platform crossing times on day 7 in the PA control group was significantly fewer than that of the CD control group (Figure 2a-2,b-2). At four weeks of age, animals in the MCPs administration groups did not show better performance than those in the PA control group, both in the training session and the test session. However, at three months of age, the average time spent in finding the platform for animals in the MCPs administration groups was significantly less than that of the PA control (Figure 2b-1), and the platform crossing times of the animals in the MCPs administration groups on the seventh day of the test was significantly more than those in the PA control group. Animals in the 1.0 g/kg and 3.0 g/kg MCPs groups paralleled the scores of the CD control group (Figure 2b-2).

### 2.3. MCPs Attenuate Neuronal Loss in the Hippocampus

Quantified Nissl histology demonstrated different neuron counts in the hippocampal CA1 area between the groups (Table 4). The mean CA1 neuron count in the PA control group was significantly decreased compared with the CD control group (*p* < 0.01), however this decrease was attenuated by 1.0 g/kg and 3.0 g/kg MCPs administration (*p* < 0.01). In contrast, Nissl-positive cells in the hippocampal CA3 and dentate gyrus (DG) areas did not reveal any significant difference between the MCPs intervention groups and the PA control group (*p* > 0.05).

**Table 4:** Effects of MCPs on neuron count in the hippocampal CA1, CA3 and DG areas (1 mm^2^) of male rats with PA.

Groups	*N*	CA1	CA3	DG
CD control	5	111.17 ± 7.111 ^††^	54.33 ± 2.503	330.75 ± 12.703
PA control	5	91.33 ± 4.844 **	48.33 ± 3.077	321.71 ± 22.874
0.33 g/kg	5	96.29 ± 3.302 **	49.14 ± 4.562	323.88 ± 10.190
1.0 g/kg	5	105.71 ± 7.158 ^††^	51.43 ± 5.062	331.38 ± 21.421
3.0 g/kg	5	106.67 ± 9.110 ^††^	53.00 ± 4.873	331.00 ± 22.829

Mean values that were significantly different from those of the CD control group: ** *p* < 0.01; Mean values that were significantly different from those of the PA control group: **^††^** *p* < 0.01.

### 2.4. MCPs Increased the Activity of Superoxide Dismutase (SOD), But Decreased the Level of Methane Dicarboxylic Aldehyde (MDA) in the Cerebrospinal Fluid of Male Rats with PA

**Figure 3:**
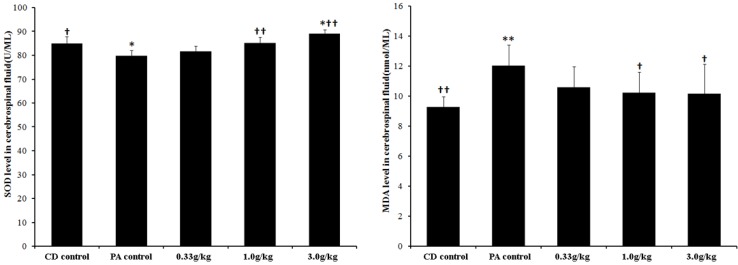
Effect of MCPs on oxidation indexes in the cerebrospinal fluid of male rats with PA (*n* = 10). Mean values that were significantly different from those of the CD control group: * *p* < 0.05, ** *p* < 0.01. Mean values that were significantly different from those of the PA control group: ^†^ *p* < 0.05, ^††^ *p* < 0.01.

As shown in Figure 3, animals with PA showed a decrease in the activity of SOD and an increase in the level of MDA in cerebrospinal fluid. Compared with the PA control group, 1.0 g/kg and 3.0 g/kg MCPs administration resulted in a decrease in MDA levels and an increase of SOD activity. No significant difference was found between the 0.33 g/kg MCPs group and the PA control group.

### 2.5. MCPs did not Change Brain/Body Weight Ratio, but Decreased Acetylcholinesterase (AChE) Activity in the Hippocampus of Male Rats with PA

**Figure 4:**
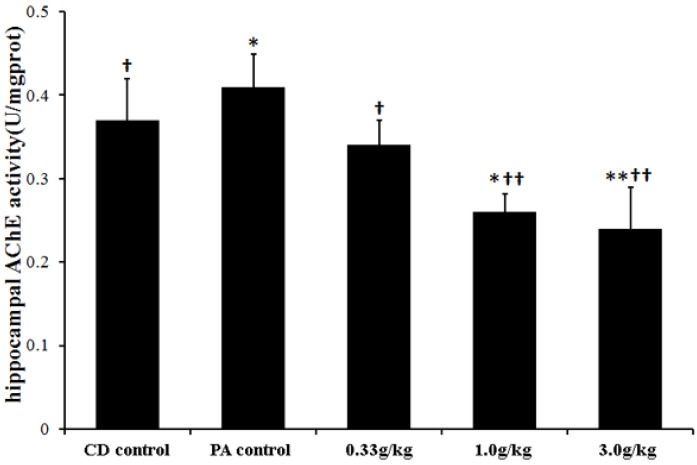
Effect of MCPs on hippocampal AChE activity in male rats with PA (*n* = 10). AChE: acetylcholinesterase. Mean values that were significantly different from those of the CD control group: * *p* < 0.05, ** *p* < 0.01. Mean values that were significantly different from those of the PA control group: ^†^ *p* < 0.05, ^††^ *p* < 0.01.

The mean brain/body weight ratios of the CD control, PA control, 0.33 g/kg MCPs, 1.0 g/kg MCPs and 3.0 g/kg MCPs groups were 0.368 ± 0.014, 0.345 ± 0.009, 0.354 ± 0.020, 0.350 ± 0.018, and 0.358 ± 0.013, respectively. No significant difference was found in the ratio of brain/body weight between the groups (*p* > 0.05). However, there was a trend that the ratio in the PA control group was lower than that of the CD control (*p* = 0.05), and 3.0 g/kg MCPs intervention groups, which appeared to show a reverse effect (*p* = 0.052 *vs.* PA control). In addition, PA resulted in an increase in hippocampal AChE activity. However, a significant decrease in AChE activity was found in all three MCPs intervention groups (Figure 4).

### 2.6. Hippocampal Expression of cAMP Response Element Binding Protein (CREB), Phosphorylated CREB (p-CREB) and Brain-Derived Neurotrophic Factor (BDNF)

**Figure 5:**
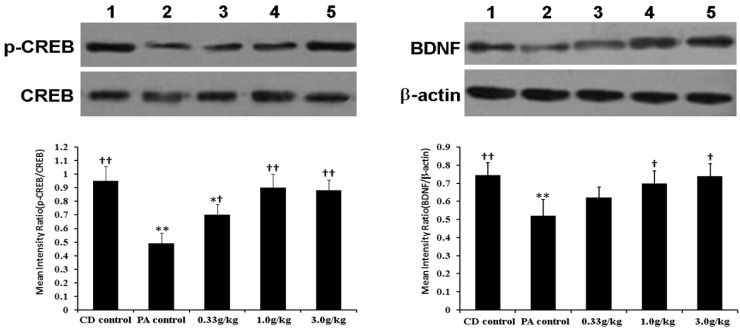
Effect of MCPs on hippocampal *CREB*, *p-CREB* and *BDNF* expression in male rats with PA 1: CD control; 2: PA control; 3: 0.33 g/kg MCPs; 4: 1.0 g/kg MCPs; and 5: 3.0 g/kg MCPs. Mean values that were significantly different from those of the CD control group: * *p* < 0.05, ** *p* < 0.01. Mean values that were significantly different from those of the PA control group: ^†^ *p* < 0.05, ^††^ *p* < 0.01.

PA inhibited the expression of BDNF, and decreased *p*-CREB/CREB ratio in hippocampus. MCPs administration reversed the expression of those proteins to some extent. The *p*-CREB/CREB ratio was significantly increased in all the three MCPs intervention groups, especially the 1.0 g/kg and 3.0 g/kg groups (*p* < 0.01), and the BDNF expression significantly increased in 1.0 g/kg and 3.0 g/kg MCPs intervention groups (*p* < 0.05) (Figure 5). 

## 3. Discussion

An asphyctic event results in reduced oxygenation of brain tissue [18]. Hypoxic damage, both in the form of necrosis and apoptosis, is evident in the cerebellum, brain stem nuclei, basal ganglia, hippocampus and cortex [19]. With regards to possible mechanisms leading to neuronal injury, excitotoxic amino acid release, cellular proteolysis, free radical generation, nitric oxide synthesis and circulating inflammatory substances, such as cytokines, have all been implicated in brain damage. To date, there is no established treatment for PA, although experimental data and clinical trials have shown that hypothermia is able to reduce death, ameliorate brain damage, and improve neurological outcomes associated with asphyxia during birth [20,21]. However, the effects of hypothermia therapy on long-lasting neurological and psychiatric consequences of PA remain unknown [20,21,22,23]. In a recent systematic review and meta-analysis of 13 clinical trials published to date, therapeutic hypothermia was associated with higher incidences of arrhythmia and thrombocytopenia in childhood [19]. 

In the present investigation, we found that MCPs, derived from enzymatic hydrolysis of chum salmon skin, were effective in improving the physiological and neurobehavioral development of male rats with PA. Theoretically, bioactive peptides can be released from dietary proteins through digestion in the gut; however, the amount of peptides is too small to induce any significant effects. In contrast, enzymatic hydrolysis of dietary proteins offers a rapid and reproducible method for the production of considerable bioactive peptide fractions, which are likely to become potential health-beneficial-food ingredients or nutraceutical preparations [10]. 

We have found in our previous study that this MCPs sample could facilitate learning and memory in aged C57BL/6J mice [17]. In the present study, PA was induced by immersing rat fetuses with uterine horns removed from ready-to-deliver rats into a water bath for 15 min. Although several other animal models had been introduced, we chose to induce asphyxia at the time when rats were delivered, for the following reasons: (i) it mimics well some relevant aspects of human delivery; (ii) it is largely non-invasive; (iii) it allows studying short- and long-term consequences of the insult in the same preparation; and (iv) it is highly reproducible among laboratories. This model implies oxygen interruption, but not any additional lesion, including vessel occlusion. Moreover, this model involves hypoxemia, acidosis, and hypercapnia, mandatory criteria for a clinically relevant model of perinatal asphyxia [24]. With this animal model, we found that PA resulted in decreased weekly body weight gain during the lactation period, which may be due to insufficient milk being obtained, caused by weakness or slow reactions. The 1.0 g/kg and 3.0 g/kg MCPs significantly increased body weight gain. This was not likely to be a result of the additional intake of amino acids, or the function of certain amino acid(s), since the PA control rats were given a 3.0 g/kg mixture of free-form amino acids, the components of which were the same as those in the MCPs. However, MCPs did not significantly improve early physiological and reflex development of rats with PA. We hypothesize that there may be some bioactive peptides in the MCPs that have better beneficial effects on neonatal growth rather than early development, which requires further investigation. However, the after weaning weekly body weight gain of the CD control group reached its peak on the third week after weaning, while it was a week later for the other groups with PA. Although 1.0 g/kg and 3.0 g/kg MCPs intervention increased the after-weaning weekly body weight gain of animals with PA, it did not change the peak timing. This may be partially related to the changes in functional maturity of the digestive system caused by PA. In the present study, we used free-form amino acids for CD control animals in order to avoid false positive errors induced by extra amino acid intake; whereas it would be better to use different processed hydrolyzed protein as control or even collagen from non-marine source in order to demonstrate whether effects related to any combinations of short peptides or specifically marine source is the major determinant for the positive effects, respectively.

The open field test at three weeks of age revealed hyperactive behavior in PA rats, which was consistent with previous studies [25,26,27]. However, Galeano *et al.* [28] and Strackx *et al.* [29], using the same model of perinatal asphyxia, but subjecting rats to 19 min of asphyxia, did not find significant differences between the experimental groups. We believe the duration of asphyxia is critical for this model, because the survival rate is drastically reduced and central nervous system damage increases with the duration of asphyxia [30,31]. It is possible that 15 min of asphyxia produce enough damage to disclose anxiety-related behaviors, but 19 min of asphyxia will induce severe damage to the brain and inhibit autonomic activity. The Morris water maze test revealed similar results. It took animals subjected to PA a significantly longer time to locate the platform than those in the CD control group, and the platform crossing times on the seventh day in PA animals were significantly decreased compared with CD controls. Performance in spatial tests, such as reference and working memory tasks in the Morris water maze, is disrupted after hippocampal damage [32]. Unfortunately, the hippocampus is one of the most affected cerebral areas following perinatal asphyxia [33,34]. Morales *et al.* reported that there was an increase in neuronal apoptosis in the hippocampus after severe perinatal asphyxia, which could explain the reduced CA1 neuron counts in the present study [35], and may be partially responsible for the poor performance of PA rats in the Morris water maze. MCPs intervention, under the present dosage, did not improve performance of PA rats in behavioral tests at four weeks of age, but significantly improved the performance at three months of age. We hypothesize that MCPs are likely to inhibit hippocampal neuron apoptosis and improve proliferation, which results in increased neuron counts in the hippocampus. However, this effect is mild and cannot compensate for the loss of neuron associated with PA over a short time. Therefore, no significant improvement can be found in early age. With longer duration of exposure to MCPs, hippocampal neuron counts, and possibly other brain cell counts, may continue to increase, further improving behavioral performance. 

Previous studies have indicated that reactive oxygen species (ROS) play a significant role in the pathophysiology of perinatal asphyxia [36]. The immature brain is particularly vulnerable to oxidative stress. In the present investigation, we found MDA significantly increased in the cerebrospinal fluid of PA animals compared with CD controls, indicating the elevated oxidation status of the brain, even at three months after PA. We hypothesize that PA caused both biochemical and molecular changes to the neonatal brain that would cause long-term, even lifelong, effects. This was confirmed by the increased activity of AChE, and decreased expression of *p*-CREB and BDNF in the hippocampus. There are several defense systems in the brain to protect against ROS, one of which is SOD. Piscopo *et al.*, using the same PA model, found that hippocampal expression of SOD was significantly increased compared with controls at post natal day (PND) 1 after PA, but decreased from PND 4 after PA. Similarly, we found in this study that SOD activity in cerebrospinal fluid was significantly decreased compared with controls at three months after PA, which was likely a result of decreased SOD gene expression in brain tissue. Recently, many peptides derived from animal or plant tissues have been found to have antioxidant functions [37,38,39]. The antioxidant peptides derived from common foods have attracted interest from pharmacists, physicians, and nutritionists for their potential therapeutic benefits. In the present study, long-term intervention with MCPs reversed the expression of antioxidant enzymes, decreased oxidation levels, and attenuated impairment of the developing brain from ROS damage, therefore may contribute to the amelioration to learning and memory impairment. We considered that the peptide fraction of sequence Glycine-Proline-Hydroxyproline (Gly-Pro-Hyp) might play an important role in the antioxidant activity of MCPs. The Gly-Pro-Hyp peptide sequence derived from fish and bovine skin has been proved to have strong antioxidative effects by Kim *et al.* [40,41]. Ao and Li [42] also found radical-scavenging effect of such peptide sequence derived from porcine collagen. The MCPs used in the present study contained relatively higher Gly, Pro and Hyp according to the analysis of amino acid composition. Therefore the Gly-Pro-Hyp peptide sequence is very likely to exist in the MCPs.

Cholinergic neurotransmission has been shown to have a range of different effects on human and animal behavior [43,44]. The activity of AChE, one of the most crucial and efficient enzymes for nerve response and function, is a functional marker for cholinergic system [45]. Recent research suggests that decreased anxiety-like behavior is directly associated with AChE activity of the hippocampus, since AChE knockdown in the hippocampus promotes anxiety-like behavior in mice [44]. In the present study, PA resulted in an increase in hippocampal AChE activity, which may partly explain the abnormal performance in the behavioral test in PA animals. In the meantime, the existence of AChE activity increase in the PA animals at three months of age also indicated that PA might result in hippocampal damage on a cellular level. The up regulation of postsynaptic AChE might be due to a lack of cholinergic transmission (degeneration of presynaptic terminal being recovering). MCPs intervention resulted in a significant decrease in AChE activity, which implied that MCPs might be able to increase cholinergic transmission by protecting presynaptic neurons from degeneration. The AChE activity reverse was also related to the improvement of behavioral performance in animals in MCPs intervention groups. 

CREB has been identified as a key molecule initiating the transcriptional activation of other genes encoding proteins that play an important role in structural and functional changes underlying information storage [46]. Indeed, CREB activation through its phosphorylation on Serine-133 (*p*-CREB) controls the induction of regulatory immediate-early genes whose products, in turn, induce the transcription of late downstream genes, and activate direct “effector” proteins, such as structural proteins, signaling enzymes or growth factors, that are essential for long-term memory. BDNF is a powerful modulator of neuronal excitability and synaptic transmission, and plays a role in hippocampal-dependent learning and memory [47,48]. We have found in our previous study that these MCPs can promote BDNF expression in the aged mouse brain [17]. Data presented herein clearly show that the expression of the above mentioned proteins decrease significantly in PA rats, but were elevated by 1.0 g/kg and 3.0 g/kg MCPs per day. Although there is no direct information about how MCPs up-regulate the expression of these proteins, there are several possible explanations: (1) MCPs have an antioxidant function, which could protect neurons and indirectly enhance protein secretion or protect the secreted proteins from oxidant damage; (2) PA may cause brain vascular damage. Collagen is known to improve vascular formation and repair, and thereby protect neuronal cells and promote the expression of BDNF.

## 4. Experimental Section

### 4.1. Preparation of MCPs

MCPs were prepared from the skin of wild-caught chum salmon (*Oncorhynchus keta*) (from the East China Sea). The preparation procedure and amino acid composition analysis of MCPs powder has been previously described [49]. The peptide purity of the MCPs sample was approximately 93.2%, and contained no carbohydrates, <0.1% fat, <2% ash content and <5% water. The molecular weight distribution of MCPs was 100–860 Da; 85.86% of which was distributed between 300 and 860 Da. The total energy was approximately 15,640 kJ·kg^−1^. The amino acid composition of the MCPs powder is shown in Table 5. 

**Table 5:** Amino acid composition of the MCPs from the skin of chum salmon.

Amino Acid	No. Residues per 100 Residues
Glycine	23.77
Glutamic acid	12.22
Proline	9.79
Hydroxyproline	7.51
Aspartic acid	7.29
Alanine	6.59
Arginine	6.08
Lysine	5.66
Leucine	4.64
Serine	4.23
Valine	2.94
Isoleucine	2.57
Threonine	2.53
Phenylalanine	2.51
Histidine	1.61
Methionine	0.03
Tyrosine	0.03

### 4.2. Animals

Healthy mature Sprague-Dawley rats of specific pathogen-free (SPF) grade were obtained from the Department of Experimental Animals of Peking University Health Science Center, Beijing, China, and were housed in temperature- and humidity-controlled rooms (23 ± 1 °C, 50%–60% humidity)with a 12 h:12 h light/dark cycle. Before experimentation, the animals were acclimatized for 3 days. Female rats were mated with healthy male rats overnight and vaginal smears were examined the next morning at 7:00 a.m. The presence of sperms in the smear signified gestational day (GD) 0. Pregnant female rats were fed a pregnancy diet and individually housed until a caesarean section was carried out. The animal protocols used in this work were evaluated and approved by the Biomedical Ethics Committee, Animal Ethic Branch of Peking University (Approval No. LA2014100). 

### 4.3. Induction of PA

Perinatal asphyxia was induced in rat pups delivered by caesarean section. In short, time-mated female rats, within the last day of gestation, were decapitated and the entire uterus was quickly removed. Immediately after hysterectomy, two fetuses were removed from the uterine horns, to be considered as caesarean-delivered controls (CD control, 0 min of asphyxia). The uterine horns containing the remaining fetuses were immersed in a water bath at 37 °C for 15 min (15 min of asphyxia). Following asphyxia, the uterine horns were incised and the pups were removed, stimulated to breathe, and after a 60-min observation period were given to surrogate dams that had given birth to healthy litters within 24 h for nursing, pending further experiments. To minimize differential rearing effects, one male caesarean-delivered pup without asphyxia (CD control), together with four male pups with 15 min of asphyxia (assigned to PA control, 0.33 g/kg MCPs, 1.0 g/kg MCPs and 3.0 g/kg MCPs intervention groups) were assigned to one foster dam (five pups per dam, for a total of 15 litters). 

### 4.4. MCPs Intervention

Pups in the MCPs intervention groups were intragastrically administered 0.33, 1.0 and 3.0 g/kg body weight MCPs dissolved in distilled water from PND 0 till 90 days of age. The pups in the CD control group and the PA control group were given, for the same period, a mixture of free-form amino acids (3.0 g/kg body weight) prepared in distilled water, which were the same as those in the MCPs sample. The amino acids mixture, rather than water vehicle, was used in the control group to avoid confounding effects caused by lower amino acid intake. The pups were individually weighted on PND 0, and then twice a week to adjust the MCPs administration volume throughout the study. The lactation period lasted 3 weeks, after which pups were weaned on PND21. 

### 4.5. Physiological Development of Pups

Each pup was monitored for the appearance of physical developmental landmarks beginning on PND 1 (pinnae detachment), PND 7 (incisor eruption), PND 12 (eyelid separation) and PND 19 (descent of testicles into the scrotum).

### 4.6. Neurobehavioral Development of Pups

Throughout the lactation period, the pups were evaluated in terms of reflex development and neuromuscular maturation.

#### 4.6.1. Surface Righting Reflex

This test measured motor function and coordination. Righting reflex on the surface was tested daily from PND 2 to PND 6. Each pup was placed on its back on a flat surface and released. The time that it took for the pup to regain all 4 paws in contact with the surface of the testing table was recorded. If righting did not occur within 60 s, the test was terminated. 

#### 4.6.2. Negative Geotaxis Reflex

The negative geotaxis reflex is an automatic, stimulus-bound orientation movement considered diagnostic of vestibular and/or proprioceptive function. The geotaxis reflex was tested from PND 3 to PND 9. Pups were placed head down on a 30° inclined plane with a rough surface, and the latency it took to turn 180° to get in a head-up position was recorded. The negative geotaxis reaction was considered successful when it was performed within 60 s and without rolling off the inclined plane.

#### 4.6.3. Cliff Avoidance Reflex

This reflex was tested from PND 3 to PND 9. The pup was manually placed with its nose and forefeet hanging over a table edge to induce a withdrawal response. The table was 30 cm above ground with a clean cotton pad (2 cm thick) positioned 5 cm below the table surface to prevent injury to the pup in case of an accidental fall off the table. The observation time was 30 s. Withdrawal of the head and forelimbs back onto the table was counted as a positive response.

### 4.7. Behavioral Tests

#### 4.7.1. Open Field Test

The open field test was carried out at PND 21. This test was used to assess the locomotion of the different rat groups. Locomotion was quantified using an open field apparatus (50 cm × 50 cm × 50 cm). The floor was divided into 25 equal squares. Each animal was placed in the center of the apparatus and observed for 5 min. The time spent in the center squares, number of squares crossed, number of rearings, and number of grooming episodes was recorded. The floor of the open field was cleaned before each testing.

#### 4.7.2. Morris Water Maze Test

This test was carried out twice in the pups, once at 4 weeks of age and again at 3 months of age. Morris water maze consisted of a circular black pool with a diameter of 120 cm and a height of 60 cm. The maze was geographically divided into four equal quadrants, and a hidden circular platform was located at the center of one of the quadrants and submerged 1.0 cm below the water. The platform remained at the same place throughout testing. The test period ran for 7 consecutive days, and was divided into two sessions: the training session in the first 6 days and the test session on the 7th day. During the training session, animals were put on the platform facing the wall for 15 s, and then each animal was tested four times per day (four start positions from each of the four quadrants). A test commenced the moment an animal was dropped in the water and finished the moment it firmly sat on the platform. A maximum of 120 s was allowed for each test. Animals that failed to locate the platform in 120 s were allowed to rest on the platform for 10 s before a new test commenced. Each test was videotaped with a camera mounted directly above the center of the pool, and the time an animal took to locate the hidden platform was recorded as the latency. On the 7th day, the platform was removed and each animal was placed into the water from the same quadrant, the number of the times it crossed the original location of the platform in 120 s was recorded.

### 4.8. Neuronal Density in the Hippocampus

After behavioral testing at 3 months of age, five animals from each group were anesthetized with pentobarbital (30 mg/kg body weight) and the brains were fixed by heart perfusion with saline, followed by 4% (w/v) paraformaldehyde in phosphate buffer solution. The brains were separated and post-fixed in the same fixative overnight for preparing frozen sections. Serial coronal frozen sections (4 μm) containing the hippocampus were obtained using a rotary microtome (HM525; MICROM; Germany), mounted on slides, and stored at −20 °C. These frozen sections were used for Nissl staining. The numbers of Nissl-positive cells in the hippocampal pyramidal cell layer (CA1 and CA3) and DG were counted under the light microscope at 400× magnification. Mean neuron count was calculated from five sections per animal.

### 4.9. Measurement of Oxidation Indexes in the Cerebrospinal Fluid

After behavioral testing at 3 months of age, the remaining animals were fasted overnight and anesthetized by intraperitoneal injection of sodium pentobarbital (30 mg/kg body weight) the next morning. A cerebrospinal fluid sample was prepared according to Wang *et al.* [50]. Briefly, a rat was placed in the prone position on a test table. A midline skin incision, approximately 2 cm in length, was made to expose the interspinous space at L6/S1. The L6/S1 interspinous ligament and S1 spinous process were carefully removed. While elevating the L6 spinous process with forceps to widen the L6/S1 interlaminar space, a 27G needle, the tip of which was bent at a 60° angle, was obliquely introduced into the subarachnoid space, and the tip of the needle was advanced in parallel with the dural sac. A sterile 1 mL syringe was used to collect the cerebrospinal fluid from the needle cup. Fifty microliters of cerebrospinal fluid was obtained within 5 min. cerebrospinal fluid samples were preserved at −20 °C until ready for analysis. SOD and MDA level were measured with commercial available kits (Nanjing Jiancheng Bioengineering Institute, Nanjing, China) according to the manufacturer’s instructions.

### 4.10. Measurement of AChE Activity in the Hippocampus

Animals were sacrificed after cerebrospinal fluid sampling at 3 months of age. The whole brain of each animal was separated immediately and weighed. The hippocampus was isolated on ice as soon as possible and preserved at −80 °C. AChE levels were analyzed with a commercially available kit (Nanjing Jiancheng Bioengineering Institute, Nanjing, China) according to the manufacturer’s instructions.

### 4.11. Western Blotting

Hippocampal expression of CREB, *p*-CREB and BDNF was detected using the Western blotting technique at 3 months of age. The hippocampus was placed into a tube with cold extraction buffer. The mixture was ultrasound blended three times for 3 s each. The slurry was left to react for 30 min at 4°C and spun at 12,000 rpm at 4 °C for 15 min. The supernatant was collected and the protein concentration was determined using the Bradford method. Samples (70 μg) were separated on a 12% SDS-polyacrylamide gel, and then electro-transferred to polyvinylidene fluoride membranes. Membranes were blocked in 5% (w/v) nonfat milk in Tris-buffered saline containing 0.05% (v/v) Tween (TBST) for 2 h, then incubated first with primary antibodies (1:1000 dilution) followed by redetection with secondary antibodies (1:1000 dilution) in TBST-5% (w/v) milk overnight at 4 °C. Detection of specific binding was performed by incubation with horse radish peroxidase-conjugated secondary antibodies (1:3000; Santa Cruz Biotechnology Inc., Santa Cruz, CA, USA) for 2 h at room temperature. Protein bands were visualized using electrochemical luminescence (Sunbio, Beijing, China). Images were scanned for densitometry, using the Quantity One software package (Bio-Rad, Hercules, CA, USA).

### 4.12. Statistics Analysis

Data was expressed as mean ± SD. SPSS 17.0 for Windows software (SPSS Inc., Chicago, IL, USA) was used for all the statistical analysis. In comparing the difference between groups, one-way ANOVA was used in analyzing all data from the open-field test, day 7 data from the Morris water maze test, and all other biochemical and molecular data; One-way repeated-measures ANOVA was used to analyze data from the first 6 days of the Morris water maze test. Statistical significance was accepted at *p* < 0.05.

## 5. Conclusions

The present study demonstrates that MCPs can facilitate long-term learning and memory in male rats with PA through reducing oxidative damage and AChE activity in the brain, and increasing hippocampus *p*-CREB and BDNF expression. Because PA is still a major pediatric issue with few successful therapies that prevent neuronal damage, the neuroprotective effect of MCPs reported here is of practical value.

## References

[1] Hack M., Fanaro A.A. (2000). Outcomes of children of extremely low birthweight and gestational age in the 1990s. Semin. Neonatol..

[2] Rapoport J.L., Addington A.M., Frangou S., Psych M.R. (2005). The neurodevelopmental model of schizophrenia: Update 2005. Mol. Psychiatry.

[3] Venerosi A., Cutuli D., Chiarotti F., Calamandrei G. (2006). C-section birth per se or followed by acute global asphyxia altered emotional behaviour in neonate and adult rats. Behav. Brain Res..

[4] Boksa P., El-Khodor B.F. (2003). Birth insult interacts with stress at adulthood to alter dopaminergic function in animal models: Possible implications for schizophrenia and other disorders. Neurosci. Biobehav. Rev..

[5] Berger R., Garnier Y. (2000). Perinatal brain injury. J. Perinat. Med..

[6] Volpe J.J. (2001). Perinatal brain injury: From pathogenesis to neuroprotection. Ment. Retard. Dev. Disabil. Res. Rev..

[7] Vannucci S.J., Hagberg H. (2004). Hypoxia-ischemia in the immature brain. J. Exp. Biol..

[8] Morales P., Bustamante D., Espina-Marchant P., Neira-Peña T., Gutiérrez-Hernández M.A., Allende-Castro C., Rojas-Mancilla E. (2011). Pathophysiology of perinatal asphyxia: Can we predict and improve individual outcomes?. EPMA J..

[9] Korhonen H., Pihlanto A. (2003). Food-derived bioactive peptides—Opportunities for designing future foods. Curr. Pharm. Des..

[10] Hartmann R., Meisel H. (2007). Food-derived peptides with biological activity: From research to food applications. Curr. Opin. Biotechnol..

[11] Miraliakbari H., Shahidi F. (2008). Antioxidant activity of minor components of tree nut oils. Food Chem..

[12] McLay R.N., Pan W., Kastin A.J. (2001). Effects of peptides on animal and human behavior: A review of studies published in the first twenty years of the journal Peptides. Peptides.

[13] Klompong V., Benjakul S., Kantachote D., Shahidi F. (2007). Antioxidative activity and functional properties of protein hydrolysate of yellow stripe trevally (*Selaroides leptolepis*) as influenced by the degree of hydrolysis and enzyme type. Food Chem..

[14] Rawat D.S., Joshi M.C., Joshi P., Atheaya H. (2006). Marine peptides and related compounds in clinical trial. Anticancer Agents Med. Chem..

[15] Aneiros A., Garateix A. (2004). Bioactive peptides from marine sources: Pharmacological properties and isolation procedures. J. Chromatogr. B Anal. Technol. Biomed. Life Sci..

[16] Chavan U.D., McKenzie D.B., Shahidi F. (2001). Functional properties of protein isolates from beach pea (*Lathyrus maritimus* L.). Food Chem..

[17] Pei X., Yang R., Zhang Z., Gao L., Wang J., Xu Y., Zhao M., Han X., Liu Z., Li Y. (2010). Marine collagen peptide isolated from Chum Salmon (*Oncorhynchus keta*) skin facilitates learning and memory in aged C57BL/6J mice. Food Chem..

[18] Du Plessis A.J., Volpe J.J. (2002). Perinatal brain injury in the preterm and term newborn. Curr. Opin. Neurol..

[19] Dell’Anna E., Chen Y., Engidawork E., Andersson K., Lubec G., Luthman J., Herrera-Marschitz M. (1997). Delayed neuronal death following perinatal asphyxia in rat. Exp. Brain Res..

[20] Azzopardi D.V., Strohm B., Edwards A.D., Dyet L., Halliday H.L., Juszczak E., Kapellou O., Levene M., Marlow N., Porter E. (2009). Moderate hypothermia to treat perinatal asphyxial encephalopathy. N. Engl. J. Med..

[21] Cebral E., Loidl C.F. (2011). Changes in neostriatal and hippocampal synaptic densities in perinatal asphyctic male and female young rats: Role of hypothermia. Brain Res. Bull..

[22] Azzopardi D., Strohm B., Marlow N., Brocklehurst P., Deierl A., Eddama O., Goodwin J., Halliday H.L., Juszczak E., Kapellou O. (2014). Effects of hypothermia for perinatal asphyxia on childhood outcomes. N. Engl. J. Med..

[23] Boutaybi N., Razenberg F., Smits-Wintjens V.E., van Zwet E.W., Rijken M., Steggerda S.J., Lopriore E. (2014). Neonatal thrombocytopenia after perinatal asphyxia treated with hypothermia: A retrospective case control study. Int. J. Pediatr..

[24] Herrera-Marschitz M., Morales P., Leyton L., Bustamante D., Klawitter V., Espina-Marchant P., Allende C., Lisboa F., Cunich G., Jara-Cavieres A. (2011). Perinatal asphyxia: Current status and approaches towards neuroprotective strategies, with focus on sentinel proteins. Neurotox. Res..

[25] Dell’Anna M.E., Calzolari S., Molinari M., Iuvone L., Calimici R. (1991). Neonatal anoxia induces transitory hyperactivity, permanent spatial memory deficits and CA1 cell density reduction in developing rats. Behav. Brain Res..

[26] Iuvone L., Geloso M.C., Dell’Anna E. (1996). Changes in open field behavior, spatial memory, and hippocampal parvalbumin immunoreactivity following enrichment in rats exposed to neonatal anoxia. Exp. Neurol..

[27] Morales P., Simola N., Bustamante D., Lisboa F., Fiedler J., Gebicke-Haerter P.J., Morelli M., Tasker R.A., Herrera-Marschitz M. (2010). Nicotinamide prevents the long-term effects of perinatal asphyxia on apoptosis, non-spatial working memory and anxiety in rats. Exp. Brain Res..

[28] Galeano P., Blanco Calvo E., Madureira de Oliveira D., Cuenya L., Kamenetzky G.V., Mustaca A.E., Barreto G.E., Giraldez-Alvarez L.D., Milei J., Capani F. (2011). Long-lasting effects of perinatal asphyxia on exploration, memory and incentive downshift. Int. J. Dev. Neurosci..

[29] Strackx E., van den Hove D.L., Prickaerts J., Zimmermann L., Steinbusch H.W., Blanco C.E., Gavilanes A.W., Vles J.S. (2010). Fetal asphyctic preconditioning protects against perinatal asphyxia-induced behavioral consequences in adulthood. Behav. Brain Res..

[30] Capani F., Saraceno G.E., Botti V., Aon-Bertolino L., de Oliveira D.M., Barreto G., Galeano P., Giraldez-Alvarez L.D., Coirini H. (2009). Protein ubiquitination in postsynaptic densities after hypoxia in rat neostriatum is blocked by hypothermia. Exp. Neurol..

[31] Loidl C.F., Gavilanes A.W., Van Dijk E.H., Vreuls W., Blokland A., Vles J.S., Steinbusch H.W., Blanco C.E. (2000). Effects of hypothermia and gender on survival and behavior after perinatal asphyxia in rats. Physiol. Behav..

[32] Cassel J.C., Cassel S., Galani R., Kelche C., Will B., Jarrard L. (1998). Fimbria-fornix *vs.* selective hippocampal lesions in rats: Effects on locomotor activity and spatial learning and memory. Neurobiol. Learn. Memory.

[33] Spasojevic S.D., Stojanovic V.D., Barisic N.A., Doronjski A.R., Zikic D.R., Babovic S.M. (2013). Neuroprotective effects of hypothermia and erythropoietin after perinatal asphyxia in newborn rats. J. Matern. Fetal Neonatal Med..

[34] Saraceno G.E., Bertolino M.L., Galeano P., Romero J.I., Garcia-Segura L.M., Capani F. (2010). Estradiol therapy in adulthood reverses glial and neuronal alterations caused by perinatal asphyxia. Exp. Neurol..

[35] Morales P., Fiedler J.L., Andrés S., Berrios C., Huaiquín P., Bustamante D., Cardenas S., Parra E., Herrera-Marschitz M. (2008). Plasticity of hippocampus following perinatal asphyxia: Effects on postnatal apoptosis and neurogenesis. J. Neurosci. Res..

[36] Morales P., Fiedler J.L., Andrés S., Berrios C., Huaiquín P., Bustamante D., Cardenas S., Parra E., Herrera-Marschitz M. (2008). Oxidative stress in perinatal asphyxia. Pediatr. Neurol..

[37] Wang B., Li L., Chi C.F., Ma J.H., Luo H.Y., Xu Y. (2013). Purification and characterisation of a novel antioxidant peptide derived from blue mussel (*Mytilus edulis*) protein hydrolysate. Food Chem..

[38] Zheng L., Su G., Ren J., Gu L., You L., Zhao M. (2010). Isolation and characterization of an oxygen radical absorbance activity peptide from defatted peanut meal hydrolysate and its antioxidant properties. J. Agric. Food Chem..

[39] Ko S.C., Kim D., Jeon Y.J. (2012). Protective effect of a novel antioxidative peptide purified from a marine *Chlorella ellipsoidea* protein against free radical-induced oxidative stress. Food Chem. Toxicol..

[40] Kim S.K., Kim Y.T., Byun H.G., Nam K.S., Joo D.S., Shahidi F. (2001). Isolation and characterization of antioxidative peptides from gelatin hydrolysate of Alaska pollack skin. J. Agric. Food Chem..

[41] Kim S.K., Kim Y.T., Byun H.G., Park P.J., Ito H. (2001). Purification and characterization of antioxidative peptides from bovine skin. J. Biochem. Mol. Biol..

[42] Ao J., Li B. (2012). Amino acid composition and antioxidant activities of hydrolysates and peptide fractions from porcine collagen. Food Sci. Technol. Int..

[43] Abreu-Villaça Y., Filgueiras C.C., Manhães A.C. (2011). Developmental aspects of the cholinergic system. Behav. Brain Res..

[44] Mineur Y.S., Obayemi A., Wigestrand M.B., Fote G.M., Calarco C.A., Li A.M., Picciotto M.R. (2013). Cholinergic signaling in the hippocampus regulates social stress resilience and anxiety- and depression-like behavior. Proc. Natl. Acad. Sci. USA.

[45] Tsim K., Soreq H. (2013). Acetylcholinesterase: Old questions and new developments. Front. Mol. Neurosci..

[46] Lamprecht R. (1999). CREB: A message to remember. Cell. Mol. Life Sci..

[47] Bimonte H.A., Nelson M.E., Granholm A.C. (2003). Age-related deficits as working memory load increases: Relationships with growth factors. Neurobiol. Aging.

[48] Gomez-Pinilla F., Huie J.R., Ying Z., Ferguson A.R., Crown E.D., Baumbauer K.M., Edgerton V.R., Grau J.W. (2007). BDNF and learning: Evidence that instrumental training promotes learning within the spinal cord by up-regulating BDNF expression. Neuroscience.

[49] Xu Y., Han X., Li Y. (2010). Effect of marine collagen peptides on long bone development in growing rats. J. Sci. Food Agric..

[50] Wang X., Kimura S., Yazawa T., Endo N. (2005). Cerebrospinal fluid sampling by lumbar puncture in rats—Repeated measurements of nitric oxide metabolites. J. Neurosci. Methods.

